# Polyphenols of Traditional Apple Varieties in Interaction with Barley β-Glucan: A Study of the Adsorption Process

**DOI:** 10.3390/foods9091278

**Published:** 2020-09-11

**Authors:** Lidija Jakobek, Ivana Buljeta, Jozo Ištuk, Andrew R. Barron

**Affiliations:** 1 Department of Applied Chemistry and Ecology, Josip Juraj Strossmayer University of Osijek, Faculty of Food Technology Osijek, Franje Kuhača 18, 31000 Osijek, Croatia; ivana.buljeta@ptfos.hr (I.B.); jozo.istuk@ptfos.hr (J.I.); 2 Department of Statistics and Data Science, Yale University, 24 Hillhouse Avenue, New Haven, CT 06511, USA; andrew.barron@yale.edu

**Keywords:** adsorption isotherm, adsorption capacity, non-linear models, interactions

## Abstract

Apple polyphenols have been studied for various beneficial bioactivities. Especially interesting are traditional, old varieties of apples for which some initial studies have suggested significant bioactivities, but they are still not completely understood. Polyphenol bioactivities can be affected by interactions with dietary fibers such as β-glucans. The aim of this study was to investigate for the first time interactions between individual polyphenols from traditional, old apple varieties (“Božićnica” and “Batulenka”) and β-glucans by studying the adsorption process. Polyphenols were extracted from the peel and flesh of traditional apples by using an ultrasonic bath and characterized with high-performance liquid chromatography. The amounts of adsorbed (*q*_e_) and un-adsorbed (*c*_e_) polyphenols were modeled with adsorption isotherms (Langmuir, Dubinin–Radushkevich, and Hill) by using improved non-linear fitting in a novel R algorithm, developed specifically for the modeling of adsorption isotherms. Polyphenols adsorbed onto β-glucan from 9 to 203 (peel, “Božićnica”), 1 to 484 (peel, “Batulenka”), 5 to 160 (flesh, “Božićnica”), and 19 to 28 mg g^−1^ (flesh, “Batulenka”). The adsorption was concentration dependent (polyphenols present in higher amount adsorbed in higher amounts). Physical sorption can be suggested. Polyphenols from traditional apples adsorb onto β-glucan and should be further studied.

## 1. Introduction

Many potential beneficial effects of dietary polyphenols have been studied [1,2,3,4]. Dietary polyphenols have been mentioned as “a promising strategy for the alleviation of autism spectrum disorder symptoms” [1]. They have the potential in reducing the risk of developing type 2 diabetes mellitus [4]. Some activities of polyphenols have remained under debate such as their chemoprevention and anti-cancer activities [3]. More studies are necessary to enhance knowledge of these potential effects. Apples and their polyphenols are particularly interesting because they are a good source of polyphenols in everyday diet. Apple polyphenols have been studied in particular for their potential beneficial effects in relieving hypoxia-induced pulmonary arterial hypertension [5] or for their therapeutic potential for obesity and related metabolic disorders [6]. Traditional, old apple varieties can be highlighted amongst many cultivars of apples that exist today. In particular, some people can develop intolerance to apples, and, in these cases, old, traditional apple varieties can be tolerated better than commercial varieties [7]. It was also shown that traditional varieties are less allergenic genotypes since they were developed before a period in which genetic improvement was conducted [8]. All of this highlights the importance of traditional apple varieties, their polyphenols, and the beneficial effects thereof.

The beneficial effects of polyphenols are connected to the amount that is available for absorption in the digestive tract in its original form: those polyphenols are bioaccessible [9]. Such bioaccessible amounts can be greatly affected by the food matrix. That is, large macromolecules from food can interact with polyphenols in upper levels of the digestive tract, thereby affecting their degradation and subsequent bioaccessibility. Amongst such molecules are dietary fibers, which are believed to reduce the degradation of polyphenols [10,11]. Earlier studies have investigated interactions between dietary fibers and polyphenols [12,13,14,15,16,17,18,19,20,21]. A review of literature on dietary fiber and polyphenol interaction is in [22]. Besides affecting the bioaccessibility of polyphenols, the interactions between dietary fibers and polyphenols can have other effects. In particular, dietary fibers can interact with polyphenols, “carrying” them to the lower parts of the digestive tract where they might be released and show potential beneficial effects, as recently reported. For instance, polyphenols transported to lower parts of the digestive tract have been shown to effect and shape gut microbiota species [2]. These processes are still not completely clear, but they highlight the importance of understanding interactions between dietary fibers and polyphenols.

The interactions between dietary fibers and polyphenols can be investigated by the in vitro adsorption process. Adsorption is a process in which molecules from liquid or gas adsorb onto the surface of an adsorbent [23,24]. The amount of molecules adsorbed (*q*_e_) and un-adsorbed (*c*_e_) can be modeled with adsorption isotherm equations [23,24], which can help describe the adsorption process. Adsorption experiments have already been used in studies of interactions between polyphenols and dietary fiber [12,13,17,19,20,21,25,26,27].

β-glucans are natural non-digestible dietary fibers [28]. Those found in cell walls of cereals and are composed of β-d-glucose with β-(1→4) linkages that form cellulose-like blocks [29], which in turn are connected with β-(1→3) linkages [28,29,30]. β-glucans can interact with polyphenols. In particular, the interaction between β-glucan and tea polyphenols [19,20] or β-glucan and total apple polyphenols [31] has been shown in some initial investigations. However, the interactions between individual polyphenols of old, traditional apple varieties and β-glucan have never been studied before. There are still many unknowns about these processes. In particular, it is not clear how individual polyphenols in the complicated systems that arise in apple extract affect interactions with β-glucan.

The aim of this study was to investigate the interactions between β-glucans and individual polyphenols from two indigenous, traditional apple varieties (“Božićnica” and “Batulenka”) by studying the adsorption process. Those apple varieties have never been studied for adsorption onto β-glucan. Moreover, to the best of our knowledge, the adsorption of individual polyphenols from apples in general has never been studied before. Adsorption data were modeled with equations of adsorption isotherms to get a better insight into the adsorption (interaction) process. For the modeling of adsorption isotherms, an improved non-linear fitting method was put to use with an algorithm in the R programming language written and developed by our group. This improved modeling gives a potential improvement in the interpretation of adsorption isotherms.

## 2. Materials and Methods

### 2.1. Chemicals

Sodium hydrogen phosphate dodecahydrate and sodium dihydrogen phosphate dihydrate were purchased from Kemika (Zagreb, Croatia). Orto-phosphoric acid (85% HPLC-grade) and methanol (HPLC grade) were from Fluka (Buchs, Switzerland) and J.T. Baker (Gliwice, Poland), respectively. Some polyphenol standards were purchased from Extrasynthese (Genay, France) (phloretin, procyanidin B1, cyanidin-3-galactoside chloride, procyanidin B2, quercetin-3-*O*-galactoside, quercetin-3-*O*-rhamnoside, and phloretin-2′-*O*-glucoside) and some from Sigma-Aldrich (St. Louis, MO, USA) ((−)-epicatechin, chlorogenic acid, (+)-catechin, quercetin, and quercetin-3-glucoside). The barley β-d-glucan was obtained from Sigma-Aldrich (St. Louis, MO, USA).

### 2.2. Apples and Polyphenol Extraction

Around 2 kg of apples was collected from local orchards (apple variety “Božićnica” from Gornji Tkalec (45°58′24.0″ N 16°27′12.0″ E) and apple variety “Batulenka” from Rude (45°46′35.6″ N 15°40′35.8″ E)). Apples of the variety “Božićnica” were peeled with a peeler, and the peel was pooled and ground in a coffee grinder. The flesh was cut, the seeds were removed, and the flesh was pooled and homogenized with a stick blender. The same procedure was repeated for the second variety “Batulenka”. Those two peel and two flesh samples were stored at −18 °C for no more than one month.

Extracts of the peel samples were made by the following procedure. Five samples of “Božićnica” peel were weighed (around 0.2 g) and added to plastic tubes. Then, 1.5 mL of 80% methanol in water was added to each tube, and the samples were vortexed. The extraction procedure was carried out by using an ultrasonic bath (RK 100, Berlin, Germany) for 15 min and then a centrifuge (Eppendorf, Hamburg, Germany) for 10 min at 10,000 rpm. The extracts from all five samples were combined, evaporated, and dissolved in 1.5 mL of methanol to get a concentrated extract of peel. One milliliter of concentrated extract was purified with Sephadex LH 20 gel, and 0.5 mL was filtered through a 0.45 µm PTFE (polytetrafluoroethylene) syringe filter and used for the determination of polyphenols in apples using the reversed-phase high-performance liquid chromatography (RP-HPLC) method. The procedure was repeated for the “Batulenka” peel. The same procedure was used for the extraction of polyphenols from the flesh of both varieties of apples.

A purification of prepared concentrated extracts of peel and flesh was carried out by using the gel chromatographic method. Sephadex LH-20 gel was prepared by adding 30 mL of 80% methanol to 5 g of gel to swell overnight and putting it in a glass column. The gel was equilibrated with 10 mL of 60% methanol and 10 mL of 10% methanol. One milliliter of concentrated extract of flesh or peel was loaded onto a column and eluted with 5 mL of 10% methanol, 5 mL of 40% methanol, 10 mL of 60% methanol, 20 mL of 80% methanol, and 20 mL of 100% methanol. The eluate after adding 80% and 100% methanol was collected, evaporated to measured volumes near 4 mL, filtered through a 0.45 µm PTFE syringe filter, analyzed on the RP-HPLC system to determine the amount of individual polyphenols, and then used for the adsorption experiment.

### 2.3. Reversed-Phase High-Performance Liquid Chromatography (RP-HPLC) Method

Polyphenols in the adsorption experiment were analyzed using an HPLC system 1260 Infinity II (Agilent technology, Santa Clara, CA, USA). The system consisted of a quaternary pump, a PDA detector (photodiode array detector) and a vialsampler. A Poroshell 120 EC C-18 column (4.6 × 100 mm, 2.7 µm) and a Poroshell 120 EC-C18 4.6 mm guard-column (Agilent technology, Santa Clara, CA, USA) were used for the polyphenol separation. The method was validated earlier in our laboratory [32]. The mobile phases were 0.1% H_3_PO_4_ (as mobile phase A) and 100% methanol (as mobile phase B). Other conditions included the flow rate of 0.8 mL min^−1^, and the injection volume of 10 µL. The gradient was 0 min 5% B, 5 min 25% B, 14 min 34% B, 25 min 37% B, 30 min 40% B, 34 min 49% B, 35 min 50% B, 58 min 51% B, 60 min 55% B, 62 min 80% B, 65 min 80% B, 67 min 5% B, and 72 min 5% B. Polyphenol standards in different concentration ranges were used for the creation of calibration curves (cyanidin-3-galactoside 0.5–24 mg L^−1^, (+)-catechin 1–48 mg L^−1^, (−)-epicatechin 1–46 mg L^−1^, procyanidin B1 0.45–22.5 mg L^−1^, phloretin-2-glucoside 1–46 mg L^−1^, phloretin 1–46 mg L^−1^, chlorogenic acid 1–46 mg L^−1^, quercetin-3-galactoside 1–49 mg L^−1^, quercetin-3-glucoside 1–45 mg L^−1^, quercetin-3-rhamnoside 1–45 mg L^−1^, and quercetin 1–43 mg L^−1^). Calibration curves were linear (r^2^ 0.9927 to 0.9998). The retention times and the UV/Vis spectrum of peaks (190–600 nm) were compared to those of authentic standards for identification. The UV/Vis spectrum of peaks agreed with the spectrum of authentic standards and their maximums were as follows: cyanidin-3-galactoside 275 and 518 nm; (+)-catechin 280 nm; (−)-epicatechin 280 nm; procyanidin B1 280 nm; phloretin-2-glucoside 282 nm; chlorogenic acid shoulder 290 nm, maximum 330 nm; quercetin-3-galactoside 255 and 355 nm; quercetin-3-glucoside 255 and 355 nm; and quercetin-3-rhamnoside 260 and 350 nm. In addition, polyphenol standards were added to the polyphenol extracts to confirm the identifications. Data from the literature [33] were used for the tentative identification of phloretin-2-xyloglucoside (UV/Vis max at 285 nm), chlorogenic acid isomer (UV/Vis shoulder at 290 nm, max at 325 nm), quercetin derivatives 1 and 2 (UV/Vis max at 255 and 355 nm), quercetin-3-xyloside (UV/Vis max at 255 and 355 nm) and quantified using phloretin, chlorogenic acid, and quercetin calibration curves. The UV/Vis maximums of all peaks are shown in Supplementary Table S1.

### 2.4. Adsorption

β-glucan (190 mg L^−1^) was dissolved in distilled water and heated (80 °C, 2 h). The reaction solution consisted of the polyphenol extract (300 µL), the β-glucan (5 mg L^−1^) and phosphate buffer (pH 5.5) to a total volume of 1 mL. The moderate pH 5.5 was chosen to avoid too low or too high pH values, which can lead to hydrolysis or oxidation reactions, respectively. Reaction solutions were mixed in a laboratory shaker (IKA KS 130, Werke, Germany) for 5 h and centrifuged at 10,000 rpm for 10 min. An aliquot (500 µL) was taken from the top of each solution and filtered through a syringe filter (0.45 µm PTFE) for the determination of un-adsorbed polyphenols using the RP-HPLC (*c*_e_ in mg L^−1^). The adsorption capacity (*q*_e_) (mg of polyphenols adsorbed onto g of β-glucan) was calculated by the mass balance equation:

(1) $$q_{e}=\frac{\left(c_{0}-c_{e}\right)V_{m}}{\gamma_{a}V_{a}}$$

where *c*_0_ is the initial polyphenol concentration in the reaction solution (mg L^−1^), *c*_e_ is the polyphenol concentration in the reaction solution at equilibrium or un-adsorbed polyphenols (mg L^−1^), *V*_m_ is the total volume of a reaction solution (L), *γ*_a_ is the β-glucan concentration (g L^−1^) and *V*_a_ is the volume of added β-glucan in a reaction solution (L). The experiment of adsorption was then repeated for more aliquots of extracts (50 to 200 µL) to get the data at more concentration levels of polyphenols. For that experiment, the *q*_e_ was calculated using Equation (1), the *c*_e_ or un-adsorbed polyphenols were determined with the RP-HPLC in mg L^−1^, but recalculated in mg. Finally, the data for *c*_e_ (mg) and *q*_e_ (mg g^−1^ β-glucan) were modeled with adsorption isotherm equations.

### 2.5. Adsorption Isotherms

Experimental data *q*_e_ and *c*_e_ (*c*_e_ in mg, *q*_e_ in mg g^−^^1^) were modeled with Langmuir, Dubinin–Radushkevich, and Hill adsorption isotherm equations. For that purpose, we used improved non-linear regression fitting methodology specifically designed for adsorption isotherms. The code for improved adsorption isotherm fitting, as described in [34], was developed as an R algorithm at Yale University by our group.

### 2.6. Statistical Analysis

Two samples of apples were studied and from each an extract of flesh and peel were obtained (four polyphenol extracts). Each extract was analyzed three times with the RP-HPLC (*n* = 3). The adsorption experiment was conducted two times for each extract at each concentration level and analyzed once on the RP-HLC (*n* = 2). Adsorption capacities were reported as means ± standard deviation (*n* = 2). The differences between adsorption capacities were studied with post hoc Tukey test using Minitab (Minitab LLC., State College, PA, USA). The *q*_e_ and *c*_e_ were modeled using adsorption isotherm equations (*q*_e_ vs. *c*_e_ diagrams) with the improved non-linear regression. The standard error (*se*) was calculated:

(2) se=(∑i=1n(qe, meas−qe, model)2)(n−a)

where *q*_e,meas_ and *q*_e,*model*_ are the *q*_e_ measured and calculated by the model, respectively, *n* represents the total number of data points, and *a* the number of parameters in the isotherm equation.

## 3. Results

### 3.1. Polyphenols and Their Adsorption Capacity

The content of polyphenols found in the peel and flesh of apples “Božićnica” and “Batulenka” is shown in Supplementary Table S1. Polyphenols in the peel belonged to subgroups of anthocyanins (cyanidin-3-galactoside), flavonols (quercetin-3-galactoside, quercetin-3-glucoside, quercetin-3-xyloside, quercetin-3-rhamnoside, and two additional quercetin derivatives), flavan-3-ols ((+)-catechin, (−)-epicatechin, and procyanidin B1), dihydrochalcones (phloretin-2′-xyloglucoside and phloretin-2′-glucoside), and phenolic acids (chlorogenic acid and chlorogenic acid isomer). Major polyphenols in the flesh were phenolic acids (chlorogenic acid and chlorogenic acid isomer) and dihydrochalcones (phloretin-2′-xyloglucoside and phloretin-2′-glucoside). The content of polyphenols was similar to that in earlier studies where traditional varieties of apples were studied [32,33].

Those polyphenols were then adsorbed onto β-glucan. The results for adsorption capacities obtained with 300 µL of polyphenol extract in the reaction solution are shown in Table 1. Polyphenols from the peel adsorbed onto β-glucan from 9 to 203 (“Božićnica”) or 1 to 484 mg g^−1^ (“Batulenka”) and polyphenols from the flesh from 5 to 160 (“Božićnica”) and 19 to 28 mg g^−1^ (“Batulenka”). It is known that the adsorbed amount depends on the initial polyphenol concentration, the pH of the environment, the buffer concentration, the temperature, and the ionic strength [12,19]. For this reason, in the literature, various amounts were reported, and it was difficult to compare these results. Some literature reported that tea polyphenols ((−)-epicatechin gallate, (−)-catechin, (−)-epigallocatechin gallate, (−)-epicatechin, and (−)-gallocatechin gallate) adsorbed onto β-glucan up to 405 mg g^−1^ [12]. Total tea polyphenols adsorbed up to 250 mg g^−1^ on β-glucan [19]. Those results were similar to our results. Plant cell wall adsorbed (+)-catechin, ferulic acid, and cyanidin-3-glucoside up to 450, 250, or 750 mg g^−1^, respectively [17]. Procyanidins were adsorbed up to 160 mg g^−1^ [25], 800 mg g^−1^ [26], ans 600 mg g^−1^ [27], on the plant cell wall. Starch nanoparticles adsorbed different polyphenols ((+)-catechin, (−)-epicatechin, (−)-epigallocatechin-3-gallate, and proanthocyanidins) in different amounts, and among them proanthocyanidins adsorbed in the higher amount, up to 200 mg g^−1^ [13]. Arabinan-rich pectic polysaccharides adsorbed up to 1800 mg g^−1^ on ferulic acid [21]. The adsorption in these studies were similar to those in our results.

**Figure 1:**
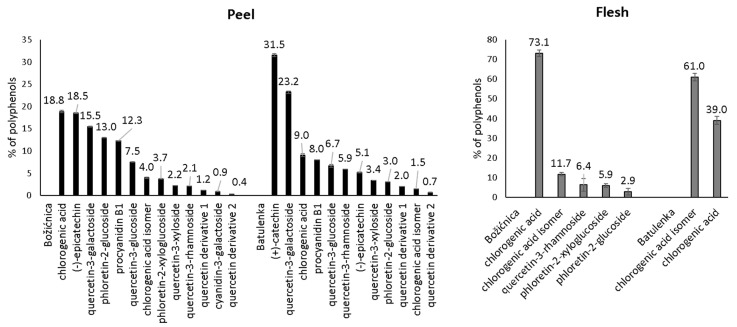
Percentage distribution of individual polyphenols in purified polyphenol extracts before adsorption (*n* = 3).

**Figure 2:**
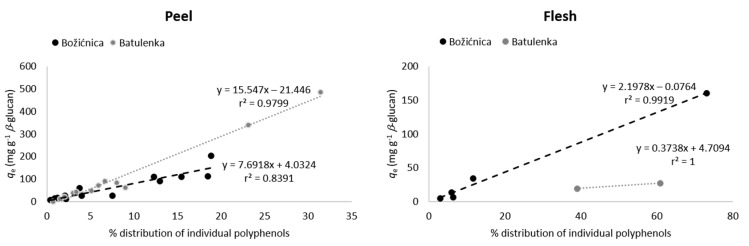
Correlation between percentage of individual polyphenols in the peel or flesh of apples and their adsorption capacities *q*_e_ (adsorption capacities obtained in the experiment with the highest volume of polyphenol extract (300 µL) in the reaction solution).

Some polyphenols from the peel can be highlighted because of their adsorption in higher amounts (Table 1): namely procyanidin B1, (−)-epicatechin, phloretin-2′-glucoside, chlorogenic acid, and quercetin-3-galactoside from “Božićnica” and (+)-catechin, quercetin-3-galactoside, and quercetin-3-glucoside from “Batulenka”. However, only for some of them that difference was statistically significant: (+)-catechin and quercetin-3-galactoside (“Batulenka”) and chlorogenic acid (“Božićnica”). A connection between the amount of polyphenols adsorbed and the amount of polyphenols present in the extract can be observed. Figure 1 shows the percentage distribution of polyphenols in the peel of both apple varieties. Compounds that adsorbed in higher amounts were actually present in higher percentage in the peel extract. To support that, the percentage of individual polyphenols in the extract and their *q*_e_ were correlated (Figure 2). The correlation was high (r^2^ = 0.84 and 0.98 for “Božićnica” and “Batulenka,” respectively).

In the flesh (Table 1), chlorogenic acid and chlorogenic acid isomer adsorbed in higher amounts than dihydrochalcones did. The higher adsorption of chlorogenic acid was statistically significant in the case of “Božićnica” flesh. Polyphenols from flesh that adsorbed in higher amounts were also present in extracts in higher percentages (Figure 1). Therefore, it can be again suggested that adsorption might be a concentration-dependent process. The correlation between percentage of individual polyphenols in the flesh and *q*_e_ is also high (r^2^ = 0.99 and 1 for “Božićnica” and “Batulenka,” respectively) (Figure 2).

### 3.2. Adsorption Isotherms

**Figure 3:**
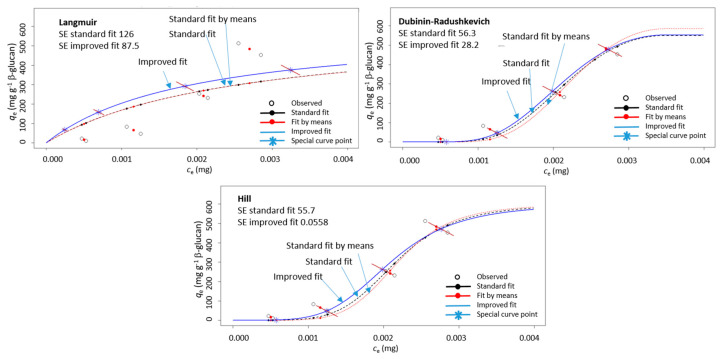
An example of a diagram showing adsorption capacity (*q*_e_) vs. un-adsorbed polyphenol amount (*c*_e_) for (+)-catechin from peel (“Batulenka”) modeled with three isotherms (Langmuir, Dubinin–Radushkevich, and Hill) using improved non-linear regression (improved fit marked with blue, standard fit with black, and standard fit of mean values with red). SE is the standard error.

The adsorption was then conducted with several different initial polyphenol concentrations and the resulting data (*q*_e_ vs. *c*_e_) were modeled with Langmuir, Dubinin–Radushkevich, and Hill isotherm equations by using the improved non-linear modeling explained in our earlier paper [34]. As described [34], in isotherm diagrams (*q*_e_ vs. *c*_e_), *q*_e_ and *c*_e_ were both measured values. They respond to the initial concentration, *c*_o_. In the case of several measurements of *q*_e_ for one initial *c*_o_, several *q*_e_ values will be positioned in the *q*_e_ vs. *c*_e_ diagram on a diagonal line (due to the mass balance equation as explained in our earlier paper [34]). In appropriate modeling, the fitted curve and point calculated by the model (*q*_e model_) should lie on that diagonal line, which is not the case in standard non-linear regression. The improved non-linear regression created in the R programming language fitted the data in a way that the calculated *q*_e model_ values by the model were on the diagonal lines. It was shown that this way of modeling created a curve with lower standard errors, which enabled more precise determination of isotherm parameters [34]. For this reason, the improved non-linear regression was chosen for the data in this study too. An example of modeling the data for *q*_e_ vs. *c*_e_ with improved non-linear regression in comparison to standard non-linear regression is shown in Figure 3. It can be seen that the improved non-linear regression gave a curve different than that of standard non-linear regression. In fact, the standard errors were smaller for the improved regression in comparison to those for the standard regression.

This showed that the curve obtained with improved modeling fits the experimental data better and by that the parameters of adsorption isotherms (Langmuir, Dubinin–Radushkevich, and Hill) can be determined with more precision. Since isotherm parameters were used for the insight into the adsorption process, precise determination of parameters can be important. This justifies the use of improved non-linear modeling of the results in this study.

#### 3.2.1. Langmuir Adsorption Isotherm

**Figure 4:**
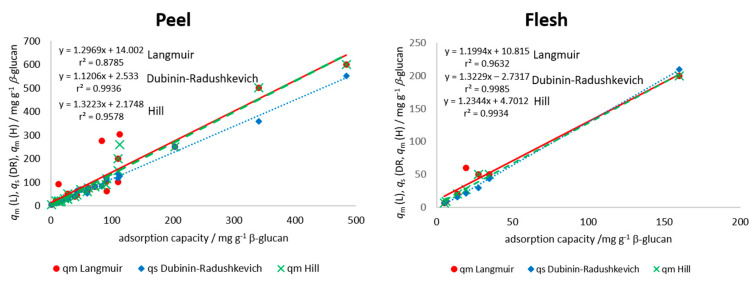
Correlation between adsorption parameters (*q*_m_ from Langmuir (L), *q*_s_ from Dubinin–Radushkevich (DR), *q*_m_ from Hill (H)), and experimental adsorption capacities (*q*_e_) for all individual polyphenols in the peel and in the flesh of apples (experimental adsorption capacities (*q*_e_) obtained in the experiment with the highest volume of polyphenol extract (300 µL) in reaction solution). Data from both types of apples are included in each correlation.

The Langmuir isotherm describes an adsorption in a monolayer on the surface of the adsorbent. The adsorbent has only a limited number of adsorption sites, and there is no interaction between adsorbed molecules [23,24,35]. It has already been used for studying polyphenol–dietary fiber interactions [12,13,19,21]. The non-linear form of the Langmuir equation is as follows:

(3) $$q_{e}=\frac{q_{m}K_{L}c_{e}}{1+K_{L}c_{e}}$$

where *K*_L_ is the Langmuir equilibrium constant of adsorption or the affinity of molecule to the adsorbent (mg^−1^) and *q*_m_ is the apparent maximum monolayer adsorption capacity of β-glucan (mg g^−1^) [23,24,35]. A plot of *q*_e_ vs. *c*_e_ was created for each polyphenol from peel and flesh, and modeling was done with the Langmuir equation using the improved non-linear regression. Parameters *K*_L_ and *q*_m_ were determined and are presented in Table 2 for the peel and in Table 3 for the flesh polyphenols. Among the polyphenols from the peel (Table 2), higher maximum theoretical adsorption capacity (*q*_m_) values were shown for procyanidin B1, (−)-epicatechin, chlorogenic acid, and quercetin-3-galactoside from “Božićnica” peel and (+)-catechin, procyanidin B1, quercetin-3-galactoside, and quercetin-3-glucoside from “Batulenka” peel. This was similar to experimentally determined adsorption capacities (Table 1). Among the polyphenols from the flesh (Table 3), higher *q*_m_ values were shown for chlorogenic acid and chlorogenic acid isomer from both apple varieties, similar to Table 1. Experimentally determined *q*_e_ and apparent theoretical values (*q*_m_) were correlated to see the agreement between the experimental values and the model parameter *q*_m_ values. A high correlation was shown (r^2^ = 0.88 for the peel and 0.96 for the flesh) (Figure 4), which suggested a good agreement between experimental data and the Langmuir parameter. These high correlations shown in Figure 4 hold even when including the values from both types of apples (different correlation lines are not needed for the two apple types). Furthermore, the differences between maximum theoretical adsorption capacities *q*_m_ of the same compound but in different extracts can be observed (for example differences in *q*_m_ for chlorogenic acid in the peel or flesh of two apples). This is the result of different initial amounts of a polyphenol in those extracts, which gave different adsorption capacities and, at the end, different isotherm parameters after modeling.

#### 3.2.2. Dubinin–Radushkevich Adsorption Isotherm

According to the Dubinin–Radushkevich isotherm, adsorption takes place on a heterogenous surface [23,35,36] and is often used to distinguish between bonds involved in the adsorption (chemical or physical adsorption). The Dubinin–Radushkevich model was chosen to see which types of bonds may be present between polyphenols and β-glucan. The ingredients of the Dubinin–Radushkevich Equation are:

(4) $$q_{e}=q_{s}exp\left(-\beta \varepsilon^{2}\right)$$

(5) $$\varepsilon =RTln\left(\frac{c_{S}}{c_{e}}\right)$$

The Dubinin–Radushkevich Equation then becomes

(6) $$q_{e}=q_{s}exp\left(-\beta R^{2}T^{2}{\left(\ln\left(\frac{c_{s}}{c_{e}}\right)\right)}^{2}\right)$$

Adsorption mean free energy can be calculated using Equation (7)

(7) $$E=\frac{1}{\sqrt{2\beta }}$$

where *q*_s_ is the theoretical isotherm saturation capacity or the maximum adsorption capacity on β-glucan (mg g^−1^); *β* is a constant related to the adsorption energy (mol^2^ J^−2^); *ε* is the Polanyi potential or adsorption potential (J mol^−1^); *R* is the gas constant (8314 J mol^−1^ K^−1^); *T* is the temperature (K); *E* is the adsorption mean free energy (J mol^−1^); *c*_s_ is theoretical saturation concentration or solubility (mg). After modeling the experimental values *q*_e_ and *c*_e_ with the Dubinin–Radushkevich equation (Equation (6)) with improved non-linear regression, parameters were determined (*q*_s_, *β*, and *c*_s_; *E* was calculated using Equation (7)). Finally, *q*_s_, *c*_s_, and *E* were reported in Table 2 and Table 3.

Among the polyphenols from the peel (Table 2), higher maximum theoretical saturation capacity values (*q*_s_) were shown for procyanidin B1, (−)-epicatechin, phloretin-2′-glucoside, chlorogenic acid, and quercetin-3-galactoside from “Božićnica” peel and (+)-catechin, quercetin-3-galactoside, and quercetin-3-glucoside from “Batulenka” peel. Among the polyphenols from the flesh (Table 3), a higher *q*_s_ was shown for chlorogenic acid (“Božićnica”). This was similar to experimentally determined adsorption capacities (Table 1). The correlation between experimental *q*_e_ and theoretical, apparent *q*_s_ was also high (r^2^ = 0.99 and 0.99 in the peel and flesh, respectively) (Figure 4), which again suggested a good agreement between experimental values and model parameter values. Furthermore, according to *E*, it might be suggested that the adsorption of polyphenols from peel and flesh was a physical process since *E* was lower than 8000 J mol^−1^ for all compounds [36,37], which suggested the creation of van der Waals forces or weak H bonds between polyphenols and β-glucan. In addition, isotherm parameters of the same compound in different extracts were different due to the different initial amounts in extracts, as mentioned earlier.

#### 3.2.3. Hill Adsorption Isotherm

The Hill isotherm describes an adsorption of different species onto a homogenous adsorbent, that allows for cooperative or non-cooperative bonding [23]. It was chosen for this study since many different polyphenols were competing for the adsorption sites. The Hill Equation is as follows:

(8) $$q_{e}=\frac{q_{m}c_{e}^{n_{H}}}{K_{D}^{n_{H}}+c_{e}^{n_{H}}}$$

where *q*_m_ is the apparent maximum adsorption capacity of β-glucan (mg g^−1^), *n*_H_ is the Hill cooperativity coefficient of the binding interaction; *K*_D_ is the Hill constant (mg). After modeling the experimental values of *q*_e_ and *c*_e_ with improved non-linear regression, the parameters *q*_m_, *n*_H_, and *K*_D_ were determined. Among the polyphenols from the peel (Table 2), higher values of maximum theoretical adsorption capacity on β-glucan were shown for procyanidin B1, (−)-epicatechin, phloretin-2′-glucoside, chlorogenic acid, and quercetin-3-galactoside from “Božićnica” peel and (+)-catechin and quercetin-3-galactoside from “Batulenka” peel. Among the polyphenols from the flesh (Table 3), a higher *q*_m_ was shown for chlorogenic acid from “Božićnica.” This was again similar with adsorption capacities (Table 1) and with *q*_m_ and *q*_s_ from Langmuir and Dubinin–Radushkevich, respectively. The correlation between experimental *q*_e_ and theoretical, apparent *q*_m_ was also high (r^2^ = 0.96 and 0.99 in the peel and flesh, respectively) (Figure 4), which again suggested a good agreement of experimental values and model parameters. Hill’s parameter *n*_H_ > 1 represents a positively cooperative bonding (when a molecule bonds to a macromolecule, other molecules can bond more easily). *n*_H_ < 1 represents a negatively cooperative bonding (when a molecule is bound to a macromolecule, the bonding of other molecules is more difficult). A non-cooperative bonding (*n*_H_ = 1), is a bonding that is independent on the molecules already bound to a macromolecule [38]. In this study, the estimated values of *n*_H_ for every polyphenol of flesh and peel were higher than 1. Accordingly, the bonding could be the result of a positive cooperation. Polyphenols bound more easily with the cooperation of other polyphenols already bound.

#### 3.2.4. Comparison of Adsorption Isotherm Models

The standard errors (SE) of Langmuir, Dubinin–Radushkevich, and Hill models are shown in Supplementary Table S2. According to the lowest standard errors, the model that fitted the best to experimental data was Hill model, followed by Dubinin–Radushkevich and then Langmuir model. This suggested that parameters obtained by the Hill model described the adsorption the best. Indeed, the Hill model fitted the results and was able to better capture the shape of curves as occurred with the Hill models with *n*_H_ > 1, appropriate to these data (Figure 3). It should be mentioned that the Dubinin-–Radushkevich model was also very good since it also captured the shape of the curve. Thus, the SE from Dubinin–Radushkevich and Hill justified the description of the adsorption by the help of these two models. Even though the Langmuir model was shown to be less appropriate for our results, all three models were able to represent the apparent maximum adsorption capacity appropriately.

## 4. Discussion

Apple polyphenols interacted with β-glucans and adsorbed in an amount that was in accordance with earlier studies [12,13,17,19,20,21,25,26,27]. Polyphenols adsorbed in a correlation with the amount present in the sample: the higher the polyphenol amount in the sample, the higher the adsorption. Since adsorption was a surface phenomenon, it was possible that polyphenols that were more abundant in the β-glucan environment made up a larger proportion along the surface of the adsorbent itself, and thus the amount of adsorbed polyphenols was higher. The fact that apple polyphenols adsorb onto β-glucan could be important for the bioactivities of polyphenols since β-glucans could be potential “carriers” of polyphenols to the lower parts of the digestive tract where polyphenols could be released and potentially show beneficial effects. Those effects should be further studied by studying the adsorption at different pH values and in simulated digestion processes. Furthermore, β-glucans can be used for developing delivery systems for apple polyphenols. β-glucans have already been studied as delivery systems for various pharmaceuticals such as doxorubicin [39], for single-strand DNA [40], for mucosal antigen [41], or even for polyphenols such as quercetin and curcumin [42]. β-glucan also showed beneficial effects itself [43], which has made it a good candidate for this purpose. Namely, it can affect enzymes and their substrates in the digestive tract as well as nutrient transportation to the places of absorption [29].

It might be suggested, given the parameters of the adsorption isotherms, that polyphenols bind to β-glucan with van der Waals interactions and maybe weak H bonds, but further studies are needed to confirm these findings. Earlier studies have shown that van der Waals forces and perhaps also weak H bonds and hydrophobic interactions are responsible for the polyphenol–dietary fiber interactions [12,19,20,25,27]. Tea polyphenols bond to β-glucan with hydrogen bonds and van der Waals interactions [12,19,20], which is similar to the suggestion of this study. Bonds between apple procyanidins and cell wall materials were H bonds and maybe hydrophobic interactions [27]. Interactions between procyanidins and cell wall material were hydrogen bonds and hydrophobic interactions [25]. Moreover, hydroxyl groups of polyphenols from apples and β-glucans probably participated in the interaction. These findings were consistent with those of previous studies [12,19,20]. It seems that interactions were cooperative, which could suggest that polyphenols already bound to β-glucan influence the binding of other polyphenols. The use of novel, improved non-linear regression for data modeling enabled more precise determination of isotherm parameters. Still, it should be mentioned that isotherm parameters should be interpreted with caution, because these are apparent, theoretical parameters. Although it would be good to study the polyphenols–dietary fiber interactions in vivo to gain true insight into the interactions, in vitro studies using adsorption process provided at least an initial insight into the mechanism of polyphenol-binding to dietary fibers.

The future research will include the studies of adsorption between apple polyphenols and β-glucan at different pH values and at different temperatures and in vitro simulated digestion processes of polyphenol–β-glucan systems.

## 5. Conclusions

Polyphenols from apples interacted with β-glucan and adsorbed onto β-glucan in a concentration dependent manner. Adsorption data can be modeled with adsorption isotherm equations. Careful interpretation of isotherm parameters allowed us to suggest that the adsorption process was a physical process with a suggestion of van der Waals forces. The adsorption could be a cooperative adsorption. More studies are needed for the interaction of polyphenols from complex real samples such as apples and β-glucan to explain those processes further. Future studies can involve kinetic studies of adsorption and the influence of different pH values and different temperatures on the adsorption.

## Figures and Tables

**Table 1:** Adsorption capacities* of β-glucan for polyphenols from peel and flesh of apples “Božićnica” and “Batulenka”.

Polyphenols	Adsorption Capacity							
	mg g^−1^							
	Peel				Flesh			
	“Božićnica”		“Batulenka”		“Božićnica”		“Batulenka”	
Anthocyanins								
cyanidin-3-galactoside	15.3 ± 0.0 ^b^							
Flavan-3-ols								
procyanidin B1	109.9 ± 10.8 ^a,b^		83.2 ± 14.9 ^c^					
(+)-catechin			483.6 ± 42.0 ^a^					
(−)-epicatechin	112.6 ± 16.0 ^a,b^		48.8 ± 2.7 ^c,d^					
Dihydrochalcones								
phloretin-2′-glucoside	91.4 ± 0.2 ^a,b^		39.0 ± 5.4 ^c,d^		5.0 ± 0.5 ^c^			
phloretin-2′-xyloglucoside	59.5 ± 2.8 ^a,b^				13.8 ± 0.7 ^b,c^			
Phenolic acids								
chlorogenic acid	203.3 ± 28.5 ^a^		61.6 ± 27.0 ^c,d^		159.7 ± 13.8 ^a^		19.3 ± 6.3 ^a^	
chlorogenic acid isomer	27.5 ± 7.5 ^a,b^		12.4 ± 2.5 ^c,d^		34.6 ± 3.8 ^b^		27.5 ± 8.8 ^a^	
Flavonols								
quercetin-3-galactoside	110.0 ± 0.5 ^a,b^		340.2 ± 44.0 ^b^					
quercetin-3 glucoside	27.6 ± 25.9 ^a,b^		90.7 ± 8.9 ^c^					
quercetin derivative 1	16.9 ± 0.5 ^b^		22.0 ± 1.6 ^c,d^					
quercetin derivative 2	8.5 ± 4.8 ^b^		1.3 ± 0.1 ^d^					
quercetin-3-xyloside	12.3 ± 11.8 ^b^		42.7 ± 4.1 ^c,d^					
quercetin-3-rhamnoside	26.8 ± 3.1 ^a,b^		71.8 ± 6.6 ^c,d^		6.3 ± 0.9 ^c^			

* Adsorption capacities from the experiment in which 300 µL of peel or flesh extract was added to the reaction solution, reported as means ± standard deviation (*n* = 2). Different letters in a column correspond to differences between polyphenols (analyzed with the post hoc Tukey test at the 0.05 significance level).

**Table 2:** Parameters of Langmuir, Dubinin–Radushkevich, and Hill adsorption isotherms obtained by improved non-linear modeling of adsorbed polyphenols from “Božićnica” and “Batulenka” peel onto β-glucan.

	Langmuir		Dubinin–Radushkevich			Hill		
	*q*_m_	*K*_L_	*q*_s_	*E*	*c*_s_	*q*_m_	*n*_H_	*K*_D_
	mg g^−1^	mg^−1^	mg g^−1^	J mol^−1^	mg	mg g^−1^		mg ^nH^
“Božićnica”								
Anthocyanins								
cyanidin-3-galactoside	20	1163	18	621	0.0007	15	12	0.0005
Flavan-3-ols								
procyanidin B1	200	117	118	1438	0.0008	200	2.1	0.0069
(−)-epicatechin	304	61	126	2545	0.0150	260	1.1	0.0121
Dihydrochalcones								
phloretin-2′-glucoside	63	291	114	499	0.0008	116	12.6	0.0071
phloretin-2′-xyloglucoside	60	1404	54	2577	0.0020	60	1.8	0.0008
Phenolic acids								
chlorogenic acid	250	175	250	3237	0.0278	250	1.8	0.0059
chlorogenic acid isomer	50	291	29	1200	0.0025	50	2.3	0.0024
Flavonols								
quercetin-3-galactoside	100	110	135	709	0.0150	150	7.6	0.0083
quercetin-3-glucoside	50	261	29	2862	0.0070	28	4.3	0.0015
quercetin derivative 1	20	1352	18	1124	0.0009	20	4.8	0.0006
quercetin derivative 2	20	1430	14	755	0.0005	20	4.2	0.0004
quercetin-3-xyloside	93	97	13	2291	0.0022	21	1.6	0.0013
quercetin-3-rhamnoside	30	1075	31	1181	0.0015	30	4.9	0.0010
“Batulenka”								
Flavan-3-ols								
procyanidin B1	276	498	83	1703	0.0008	84	3.8	0.0003
(+)-catechin	600	515	553	1144	0.0035	600	4.7	0.0021
(−)-epicatechin	70	849	70	738	0.0010	70	5.5	0.0008
Dihydrochalcones								
phloretin-2′-glucoside	40	7820	41	1688	0.0004	40	3.9	0.0002
Phenolic acids								
chlorogenic acid	75	1231	74	501	0.0015	75	4.8	0.0008
chlorogenic acid isomer	18	3594	18	459	0.0002	18	12.7	0.0002
Flavonols								
quercetin-3-galactoside	500	526	359	1580	0.0025	500	2.7	0.0017
quercetin-3-glucoside	100	1225	119	769	0.0010	90	9.9	0.0007
quercetin derivative 1	30	1553	25	393	0.0005	30	12.6	0.0004
quercetin derivative 2	5	809	5	299	0.0005	5	8.6	0.0004
quercetin-3-xyloside	45	2125	63	638	0.0006	45	10.2	0.0005
quercetin-3-rhamnoside	80	976	80	559	0.0012	80	10.7	0.0009

Adsorption capacities were measured two times for each concentration level of polyphenols, modeled with improved non-linear regression. *q*_m_ is the apparent maximum monolayer adsorption capacity of β-glucan; *K*_L_ is the Langmuir equilibration constant of adsorption; *q*_s_ is the theoretical isotherm saturation capacity or the maximum adsorption capacity of β-glucan; *E* is the adsorption mean free energy; *c*_s_ is the theoretical saturation concentration or solubility; *n*_H_ is the Hill cooperativity coefficient; *K*_D_ is the Hill constant.

**Table 3:** Parameters of Langmuir, Dubinin–Radushkevich, and Hill adsorption isotherms obtained by improved non-linear modeling of adsorbed polyphenols from “Božićnica” and “Batulenka” flesh onto β-glucan.

	Langmuir		Dubinin–Radushkevich			Hill		
	*q*_m_	*K*_L_	*q*_s_	*E*	*c*_s_	*q*_m_	*n*_H_	*K*_D_
	mg g^−1^	mg^−1^	mg g^−1^	J mol^−1^	mg	mg g^−1^		mg ^nH^
“Božićnica”								
Dihydrochalcones								
phloretin-2′-xyloglucoside	20	3310	15	998	0.0003	20	3.9	0.0002
phloretin-2′-glucoside	6	10,890	6	687	0.0001	6	10.4	0.00009
Phenolic acids								
chlorogenic acid	200	220	209	655	0.0047	200	9.6	0.0034
chlorogenic acid isomer	50	1473	43	761	0.0004	50	6.1	0.0005
Flavonols								
quercetin-3-rhamnoside	9	1634	9	355	0.0006	9	47	0.0006
“Batulenka”								
Phenolic acids								
chlorogenic acid	60	928	21	2064	0.0006	27	2.2	0.0003
chlorogenic acid isomer	50	1002	29	1584	0.0008	50	2.3	0.0007

Adsorption capacities were measured two times for each concentration level of polyphenols, modeled with improved non-linear regression. *q*_m_ is the apparent maximum monolayer adsorption capacity of β-glucan; *K*_L_ is the Langmuir equilibration constant of adsorption; *q*_s_ is the theoretical isotherm saturation capacity or the maximum adsorption capacity of β-glucan; *E* is the adsorption mean free energy; *c*_s_ is the theoretical saturation concentration or solubility; *n*_H_ is the Hill cooperativity coefficient; *K*_D_ is the Hill constant.

## Supplementary Materials

The following are available online at https://www.mdpi.com/2304-8158/9/9/1278/s1, Table S1. UV/Vis spectral characteristics of phenolic compounds in the peel and flesh of “Božićnica” and “Batulenka” apples and their content. Table S2. Standard errors of adsorption isotherm models fitted with improved non-linear regression of *q*_e_ and *c*_e_ for each polyphenol compound from peel and flesh of apples “Božićnica” and “Batulenka.”
