# Supplementary material for: Polyphenols of Traditional Apple Varieties in Interaction with Barley β-Glucan: A Study of the Adsorption Process
Source: Foods. 2020 Sep 11;9(9):1278. doi: 10.3390/foods9091278 (PMC7556014; doi:10.3390/foods9091278)
Supplement: Supplementary file 1 [file foods-09-01278-s001.pdf]

Table S1. UV/Vis spectral characteristics of phenolic compounds in the peel and flesh of 'Božićnica' and 'Batulenka' apples and their content

	λ_{max} nm	Phenolic compound content* mg kg^{-1} FW			
		PEEL		FLESH	
		Božićnica	Batulenka	Božićnica	Batulenka
**anthocyanins**					
cyanidin-3-galactoside	275, 518	35.5 ± 1.0			
**flavan-3-ols**					
procyanidin B1	280	50.3 ± 0.4	2.2 ± 0.0		
(+)-catechin	280		175.1 ± 5.4		
(-)-epicatechin	280	44.3 ± 7.6	16.5 ± 0.1		
**dihydrochalcones**					
phloretin-2-xyloglucoside	285	117.0 ± 0.7		10.0 ± 3.4	
phloretin-2-glucoside	282	140.6 ± 3.1	7.3 ± 0.2	7.8 ± 5.8	
**phenolic acids**					
chlorogenic acid	sh 290, max 330	169.6 ± 3.4	46.6 ± 1.4	123.0 ± 20.5	17.9 ± 1.1
chlorogenic acid isomer	sh 290, max 325	37.2 ± 1.5		30.7 ± 6.1	29.5 ± 0.6
**flavonols**					
quercetin-3-galactoside	255, 355	203.0 ± 5.5	102.4 ± 1.1		
quercetin-3-glucoside	255, 355	54.0 ± 2.9	27.9 ± 1.8		
quercetin derivative 1	255, 355	17.0 ± 0.4	8.4 ± 0.2		
quercetin derivative 2	255, 355	3.2 ± 0.0	1.4 ± 0.0		
quercetin-3-xyloside	255, 355	37.9 ± 1.7	16.8 ± 0.4		
quercetin-3-rhamnoside	260, 350	20.6 ± 1.1	21.3 ± 0.5	3.3 ± 0.1	
		930.2 ± 29.3	425.9 ± 11.1	174.8 ± 35.9	47.4 ± 1.7

sh - shoulder

*phenolic compound content in extracts before purification and before adsorption

Table S2. Standard errors of adsorption isotherm models fitted with improved non-linear regression of q_e and c_e for each polyphenol compound from peel and flesh of apples ´Božićnica´ and ´Batulenka´

	Langmuir	Dubinin-Radushkevich	Hill
**PEEL**			
		**'Božićnica'**	
cyanidin-3-galactoside	4.71	3,59	0.24
procyanidin B1	52.80	58.00	0.57
(-)-epicatechin	60.20	59.70	0.60
phloretin-2'-glucoside	36.20	3.96	0.04
phloretin-2'-xyloglucoside	8.79	7.45	0.07
chlorogenic acid	29.90	0.23	0.24
chlorogenic acid isomer	5.62	5.00	0.04
quercetin-3-galactoside	36.20	14.50	0.15
quercetin-3 glucoside	18.80	18.60	3.29
quercetin derivative 1	4.52	1.44	0.01
quercetin derivative 2	2.82	2.61	0.02
quercetin-3-xyloside	6.41	1.41	1.41
quercetin-3-rhamnoside	6.71	2.59	1.11
		**'Batulenka'**	
procyanidin B1	12.00	1.57	1.56
(+)-catechin	87.50	28.20	0.06
(-)-epicatechin	12.60	4.97	0.04
phloretin-2'-glucoside	7.51	2.84	0.07
chlorogenic acid	20.80	16.90	0.21
chlorogenic acid isomer	4.14	1.54	0.11
quercetin-3-galactoside	49.80	22.40	0.20
quercetin-3 glucoside	25.10	4.67	0.06
quercetin derivative 1	6.96	0.76	0.03
quercetin derivative 2	036	0.37	0.00
quercetin-3-xyloside	12.50	2.77	0.02
quercetin-3-rhamnoside	21.30	3.08	0.04
**FLESH**			
		**'Božićnica'**	
phloretin-2'-glucoside	1.23	0.82	0.81
phloretin-2'-xyloglucoside	2.84	1.59	0.01
chlorogenic acid	45.40	12.10	0.12
chlorogenic acid isomer	8.12	4.53	0.04
quercetin-3-rhamnoside	1.44	0.71	0.09
		**'Batulenka'**	
chlorogenic acid	3.68	1.21	1.21
chlorogenic acid isomer	6.45	4.57	0.05
